# Can Inner Experience Be Apprehended in High Fidelity? Examining Brain Activation and Experience from Multiple Perspectives

**DOI:** 10.3389/fpsyg.2017.00043

**Published:** 2017-01-27

**Authors:** Russell T. Hurlburt, Ben Alderson-Day, Charles Fernyhough, Simone Kühn

**Affiliations:** ^1^Department of Psychology, University of NevadaLas Vegas, NV, USA; ^2^Department of Psychology, Durham UniversityDurham, UK; ^3^Center for Lifespan Psychology, Max Planck Institute for Human DevelopmentBerlin, Germany; ^4^Department of Psychiatry and Psychotherapy, University Clinic Hamburg-EppendorfHamburg, Germany

**Keywords:** descriptive experience sampling (DES), fMRI, pristine inner experience, fidelity, introspection

## Abstract

We discuss the historical context for explorations of “pristine inner experience,” attempts to apprehend and describe the inner experiences that directly present themselves in natural environments. There is no generally accepted method for determining whether such apprehensions/descriptions should be considered high fidelity. By analogy from musical recording, we present and discuss one strategy for establishing experiential fidelity: the examining of brain activation associated with a variety of experiential perspectives that had not been specified at the time of data collection. We beeped participants in an fMRI scanner at randomly-determined times and recorded time-locked brain activations. We used Descriptive Experience Sampling (DES) to apprehend and describe the participant's experience that was ongoing at each beep. These apprehensions/descriptions were obtained with no specific theoretical perspective or experimental intention when originally collected. If these apprehensions/descriptions were of high fidelity, then these pairings of moments of experience and brain activations should be able to be productively examined and re-examined in multiple ways and from multiple theoretical perspectives. We discuss a small set of such re-examinations and conclude that this strategy is worthy of further examination.

## Introduction

A main theme (if not *the* main theme) of the (roughly 135 year) history of psychology is what science should do about first-person reports of experience. The first third (roughly 1879-1925) of psychology's history was marked by Introspection (spelled, as is usual for that period, with a capital *I* to call attention to the systematic methodological characteristics of the investigations conducted by Wundt, Titchener, Külpe, and others), as psychology attempted to study directly the elements of consciousness. However, as is well-known, the several Introspection laboratories failed to agree on fundamentally important issues such as whether imageless thought existed (Lieberman, 1979). That disagreement left the Introspectionists vulnerable to vitriolic attack from all sides: psychoanalysts held that important processes were unconscious and therefore by definition non-introspectionable; behaviorists held that neither conscious nor unconscious contents were publicly observable and therefore should be excluded from science; the rising interest in individual differences undermined the Introspectionists' search for universal mental elements. For those and other historical or systemic reasons (Danzinger, 1980), by about 1925 Introspection as psychological method had gone down in flames.

Psychological history's second third (1925-1970) was marked by the suppression of introspection. The behaviorists, broadly speaking, had gained control of psychological science. Introspection was so thoroughly discredited that the term was never even mentioned in psychological-method textbooks in this period except as the target of a historical condemnation (Hurlburt et al., 2006). Explorations of private experience largely disappeared from psychological science and mention of the word “consciousness” became rare. However, it gradually seemed to become apparent that private experiences (thoughts, feelings, etc.) were fundamentally important features of the human condition and that their radical exclusion by psychological science was too extreme.

As the behaviorists lost their dominating grip, psychological history's third (1970-present) saw a resurgence in psychological investigations of the human aspects such as thinking, feeling, self-concept, and so on, that had been excluded during behaviorism's suppression. Psychology became “cognitive,” interested in mind, mental contents, and mental processes (with textbook titles like *Cognitive Psychology: Connecting Mind, Research and Everyday Experience* (Goldstein, 2014) and *Cognition: Exploring the Science of the Mind* (Reisberg, 2015). Psychological investigations were often performed using casual and untrained introspection (now written with a lower-case *i* to contrast it with the formal Introspection of the first third of psychology, and often called “self-report”) that presumed that people had straightforward access to their mental processes. However, these new introspections were soundly criticized, for example, by Nisbett and Wilson (1977), who concluded in a widely cited review that

the accuracy of subjective reports is so poor as to suggest that any introspective access that may exist is not sufficient to produce generally correct or reliable reports (Nisbett and Wilson, 1977, p. 233).

The behaviorists continued their criticism. Skinner, for example, criticized mentalistic explanations of behavior:

I see no evidence of an inner world of mental life relative either to an analysis of behavior as a function of environmental forces or to the physiology of the nervous system…. The appeal to cognitive states and processes is a diversion which could well be responsible for much of our failure to solve our problems (Skinner, 1977, p. 10).

Modern psychology has not resolved these criticisms, resulting in a deep ambivalence about whether first-person reports should be admitted as psychological data (Wooffitt and Holt, 2011). Hurlburt and Heavey (2001) called it a chasm. On the one side are those who, following the behaviorists, hold that introspection is impossible—that first-person reports of inner experience cannot be trusted and should continue to be excluded from scientific consideration. Instead of asking people to describe directly their mental processes, these investigators infer mental characteristics based on the observation of non-introspective measures such as reaction time, eye movements, and brain activity.

On the other side of the chasm are those who hold, Skinner and Nisbett/Wilson notwithstanding, that introspection is necessary, that first-person accounts reveal important characteristics of people (and are even essential in understanding psychopathology), and that first-person accounts are acceptable within science. These psychologists aim directly at inner experience, typically asking participants to fill out questionnaires that enquire, for example, about: their experiences while having undergone a resting state acquisition in a magnetic resonance imaging (MRI) scanner; the frequency of their rumination; the frequency of their obsessive thoughts; their ability to maintain self-worth; the characteristics of their inner speech, of their non-judgmental mindfulness, or of their attitude toward political involvement; or any of thousands of other supposedly experiential features. These questionnaire reports are validated in a variety of ways (e.g., by correlating with existing questionnaires), often under the (usually unstated) belief that establishing validity implies that the observations themselves are of adequate fidelity.

Hurlburt and Heavey (2001) held that both sides of the chasm deserve implementation. As the behaviorally inclined suggest, modern psychology should profit from the painful lessons of Introspection's calamitous demise, as re-articulated by Skinner (1977) and by Nisbett and Wilson (1977): there is indeed good reason to distrust self-reports (Hurlburt and Heavey, 2015). At the same time, however, **inner experience** is indeed a defining aspect of the human condition, and psychological science must use first-person reports of inner experience—inner experience cannot be adequately inferred from external measurements like reaction time. On that view, psychological science should wrestle to the ground the question: under what conditions should first-person reports be held to be high fidelity accounts of inner experience? However, rather than address the question, the two sides of the chasm have largely gone their separate ways. Many cognitive psychologists continue to downplay first-person reports, whereas others continue to rely on first-person questionnaires with little regard for the critiques from the other side.

KEY CONCEPT 1 **Inner experience**

The phenomena (including seeings, hearings, inner speakings, thoughts, tickles, sensations, feelings, etc.) that naturally present themselves as ongoing and as directly apprehended (as “before the footlights of consciousness”) at particular moments. Includes apprehensions of the external and internal environments.

## Descriptive experience sampling

This historical sketch, like any 1000-word sketch of a 135-year period, is an oversimplification, and one could quibble about the dates and so on. However, it sets the context for the explorations of “**pristine** inner experience” (Hurlburt and Akhter, 2006) undertaken by Hurlburt and his colleagues, who have sought to honor both sides of the first-person-report chasm by suggesting that pristine experiences, the ongoing naturally occurring thoughts, feelings, sensations, and so on that appear directly “before the footlights of consciousness” (as James, 1890/1952, p. 153 would say), are characteristically human experiences that deserve to be considered by psychological science. Hurlburt and his colleagues have advanced **Descriptive Experience Sampling (DES)** method (Hurlburt, 2011; Hurlburt and Akhter, 2006; Hurlburt and Heavey, 2006), which is an attempt at a procedure that **apprehends** and describes pristine inner experience in high fidelity. DES uses a random beeper to signal participants to attend to their ongoing experience at the moment of the beep, and coaches them in so doing using an **iterative** procedure (Hurlburt, 2009, 2011). They have argued that DES's exploration of pristine inner experience avoids the pitfalls that led to Introspection's demise by focusing on experience itself rather than searching for elements that underlie experience (Monson and Hurlburt, 1993). Furthermore, DES avoids the traps of mentalism and language limitations described by Skinner (Hurlburt and Heavey, 2001). And furthermore, Hurlburt and Heavey (2001) note that Nisbett and Wilson specifically exempted DES-type investigations from their condemnation of introspection:

We also wish to acknowledge that the studies do not suffice to show that people *could never* be accurate about the processes involved. To do so would require…theoretically interesting procedures such as interrupting a process at the very moment it was occurring, alerting subjects to pay careful attention to their cognitive processes, coaching them in introspective procedures, and so on (Nisbett and Wilson, 1977, p. 246).

(Further discussion of Nisbett and Wilson's critique and psychology's over-generalization of it is in Hurlburt and Schwitzgebel, 2007, and in Hurlburt and Heavey, 2001).

KEY CONCEPT 2 **Pristine**

As it naturally occurs, undisturbed by the act of observation, not intentionally manipulated. Pristine inner experiences are intimately personal, produced of, by, and for the individual in the individual's own manner. They can be simple or complex, clean or messy. Pristine connotes natural and *not* specifically altered; it does not connote clean or pure (a pristine forest is often mucky or brutal).

KEY CONCEPT 3 **Descriptive experience sampling (des)**

An attempt at apprehending and describing pristine inner experience in high fidelity. Using a random beeper in natural environments, it explores ongoing-at-the-moment-of-the-beep experience; an “expositional” interview then explores and describes the experience. It is an evolving method and set of principles aimed at submitting to the constraints that the exploration of experience imposes.

KEY CONCEPT 4 **Apprehends**

To grasp or make thematic some aspect or aspects of ongoing inner experience. At any moment there are ongoing a welter of stimuli (inner and outer, visual, auditory, imaginary, etc.) to which people are skillfully responsive (not necessarily thematically). Out of that welter emerge one or a few directly apprehended experiences; those are the pristine inner experiences.

KEY CONCEPT 5 **Iterative**

The incremental acquisition of apprehensional and descriptive skill. DES holds that all explorations of inner experience should be assumed to begin at low fidelity or worse, and therefore obtaining high fidelity descriptions of inner experience requires a method that trains successive approximations that ultimately may become satisfactorily close to high fidelity.

Hurlburt (2011, chapter 17) made the provocative claim that pristine inner experience is radically non-subjective—that is, it is not the result of opinion or impression but instead is directly apprehendable, as Skinner and the behaviorists required—and has defended the adequacy of DES against skeptics, as in Hurlburt and Schwitzgebel (2007) and in Caracciolo and Hurlburt (2016). If such claims and defenses are at least partially correct, then science may have a way forward that escapes the experiential chasm. Science, however, has yet to determine a way to evaluate such claims; this paper is intended as a contribution.

## Pristine experience

As defined by Hurlburt (2011) and Hurlburt and Akhter (2006), pristine inner experiences are phenomena (including thoughts, feelings, sensations, perceptions, etc.) that directly present themselves as we navigate our way through our natural environments. We spend our waking lives immersed in our own experiences, so it might seem that we have privileged or infallible access to our own pristine experience, but Hurlburt (2011) argued that people are generally mistaken, and often grossly mistaken, about the characteristics of their own pristine experience. For example, Baars held that inner speech is ubiquitous (e.g., “Human beings talk to themselves every moment of the waking day;” Baars, 2003, p. 106), whereas DES investigations suggest that many people talk to themselves never or almost never (Hurlburt et al., 2013; Hurlburt and Heavey, 2015; cf. Alderson-Day and Fernyhough, 2015).

Let us consider a few samples of pristine experience from “Susan,” a participant in the resting-state functional MRI (fMRI) study by Hurlburt et al. (2015). For now, let's assume that this is a high-fidelity description of Susan's pristine experience—we will return to the assumption of high-fidelity below.

1:52:12 pm (sample 5.1): [Susan is lying quietly in the MRI scanner while a resting state acquisition is being made.] At the moment of the beep she is visualizing very strongly a scene from yesterday: she clearly innerly sees her boyfriend and his mother on a hillside next to the lake [much like she had actually seen them yesterday]. She sees the boyfriend in the shade, his mother in the sun, and (blurrily) a sea of people around them. [Before the beep she had been thinking that they look like monkeys, the way monkeys perch in family groups.] Simultaneously she is somehow saying to herself in her own voice something like “they *do* look like monkeys.” These are words floating around but they don't make a full sentence. This is something like implied words rather than actually experienced words.

*Pristine experience* refers to a phenomenon that at a particular moment appears directly “before the footlights of consciousness.” At the moment of this beep, Susan's pristine experience includes (a) innerly seeing her boyfriend and his mother and (b) the inner incompletely worded saying of something like “they *do* look like monkeys.” Pristine experience does *not* include anything that is not directly experienced. It therefore does not include aspects of the current context (e.g., that Susan was lying in the scanner at the Max Planck Institute in Berlin or the sensation of the scanner stretcher against her back) unless those aspects are directly apprehended; it also does not include historical facts (e.g., that yesterday Susan was at the lake) unless somehow that fact is at the moment directly apprehended; nor does it include impressions (e.g., that mother and son are co-dependent) unless somehow that impression is at the moment directly apprehended. Pristine experience does not include putative causation (e.g., that Susan innerly sees them *because* she thinks they are co-dependent) unless that causation is at the moment directly apprehended; and it does not include putative personality characteristics (e.g., that Susan is an introvert). DES calls that nearly infinite list of potential experiences the “welter” (Hurlburt and Heavey, 2006). That is, out of all the things that could conceivably have become part of Susan's experience at 1:52:12 (out of the welter of potentialities), Susan did—for whatever reason—experience the inner seeing of the boyfriend, mother, and hillside and the inner speaking of the incompletely worded “they *do* look like monkeys.”

Distinguishing between pristine experience and all else is of fundamental importance to the science of experience because pristine experience (but not those alternatives) is radically non-subjective (Hurlburt, 2011, ch. 17). Susan's pristine experience was private (available only to her), to be sure, and cannot be directly verified by an external observer (who at 1:52:12 would see only that Susan was lying quietly in the scanner). However, at 1:52:12, whether Susan was or was not innerly seeing her boyfriend and his mother is *not* a matter of subjective impression but of (Susan's radically non-subjective) direct apprehension. By contrast, an impression of mother-son co-dependence (an example from the welter of non-pristine alternatives) is not directly apprehended at any given moment—co-dependence is not apprehended but inferred, and that inferential process might on other occasions lead to the mother's overbearingness, or to the boyfriend's weakness, or to any of a host of more-or-less related constructs. That is, co-dependence does not have the “either it was or was not” characteristic that pristine experience has.

And even if Susan's co-dependence impression could be established, it would be difficult if not impossible to establish the extent to which Susan's version of co-dependence is similar to that of others (see Skinner, 1974, and its discussion in Hurlburt and Heavey, 2001). By contrast, we can interrogate Susan about what she means when she says she “sees” her boyfriend at 1:52:12. In what ways is this “seeing” similar to or different from seeing the pencil there on the table? In what ways is this “seeing” similar to or different from hearing Elton John's “Candle in the Wind” playing through your earbuds? In what ways is this “seeing” similar to or different from tasting the chocolate candy you are eating? With further questioning, it turns out that Susan's inner seeing is experientially much more similar to external seeing than it is to external hearing or tasting. That kind of refinement is not possible for the co-dependence impression (see Hurlburt and Heavey, 2001), or the causation inference, or many of the other potentialities in the welter.

We can (and did) perform that kind of refinement in the interview about the inner speaking of “they *do* look like monkeys.” We discovered that it was similar to speaking aloud in that it was in Susan's own voice. However, we discovered, with Susan, and surprisingly to Susan herself, that the words were not clearly defined. We discovered that there were words involved in this experience (not merely the meanings that might be intended); somehow Susan was *saying* something like these words (that is, the experience was of *speaking*, not of seeing the words, not of merely knowing that the words were present). We discovered that this description of not-clearly-defined-words was not merely an artifact of Susan's rhetorical style (or of our way of interviewing), because at other samples Susan's descriptions of words were unambiguously detailed…

2:14:29 pm (sample 5.3): Susan is looking at her eyes in the scanner mirror, noting the distance from her eyes to her eyebrows. She is saying to herself, “Wish I looked like that standing,” in her own soft inner voice with a slightly ironic or humorous tone. This is a completely worded sentence except that the subject “I” is implied rather than explicitly spoken; the words and their manner of presentation (slightly ironic or humorous tone) are unambiguously apprehended. Simultaneously, Susan is beginning to attend to the symmetry of her eyes/eyebrows, but that is not (or perhaps not yet) a complete thought.

2:19:02 pm (sample 5.4): [Susan had been wondering whether the pitch in which she speaks correlates with how she feels.] At the moment of the beep she was innerly speaking, answering that question: “I think so,” but with the intonation of the word “think” expressing uncertainty.

…whereas, at other samples, Susan's thinking involved no words at all:

11:08:44 am (sample 8.8) Susan is innerly seeing her best friend Angie slightly in profile (with Angie's pony tail on the right and farther away). Susan clearly sees Angie's face and hair but not what she is wearing. Susan knows that Angie is standing in front of a café or somewhere in her hometown, although the background is blurry. At the moment of the beep Susan is also thinking/wondering, wordlessly, something like *Will we remain besties?* while simultaneously feeling love for her and missing her. This feeling is a warmth transferring from Susan to Angie; this is a mental not physical warmth.

As a result, the DES method applied in Hurlburt et al. (2015) would conclude that Susan's apprehensions of her pristine experiences include a range of completeness in the inner expression of words, ranging from quite completely expressed with explicitly apprehended prosody (5.3) to innerly speaking with implied words (5.1) to thinking without words at all (8.8).

## Establishing fidelity

The method applied in Hurlburt et al. (2015) would further conclude that Susan's range of completeness was a characteristic of her pristine inner experience, not merely an artifact of the data acquisition or interview process. That is, Hurlburt et al. claimed that they had provided high **fidelity** descriptions of Susan's (and their other participants') pristine inner experiences. Whether such a claim should be believed lies in the center of the chasm described above. If DES (or some other method) actually provides high fidelity apprehensions and descriptions of pristine inner experience, then there is a way out of the chasm: the behaviorally inclined can require apprehensions/descriptions whose fidelity is credible, and the experientially inclined can acknowledge that pristine inner experience gives a glimpse into the human condition. Currently, however, there is no well-developed scientific strategy to evaluate a claim about the fidelity of apprehensions/descriptions of private experience (Price and Barrell, 2012). Many would claim that because inner experience is private, it and descriptions thereof cannot *possibly* be of high fidelity. We begin with a thought experiment.

KEY CONCEPT 6 **Fidelity**

The degree to which an apprehension or a description of pristine inner experience faithfully reflects, genuinely conveys, and non-misleadingly suggests important features of that pristine inner experience. Fidelity's hallmarks are lack of distortion, misrepresentation, exaggeration, avoidance. Fidelity is not perfection, nor is it accuracy; it is straightforwardness and reflection.

Suppose you are a deaf recording engineer, and you have before you a recording of a symphony, its score, and some sophisticated audio editing equipment. You wish to know whether the recording is of high fidelity. You decide to examine the recording from the perspective of oboes: the score tells you that oboes should be playing at measures 21, 57, 63…, and not playing at measures 14, 43, 67…. You know something about the timbre (that is, the wave form) of oboes; you use your equipment and discover that there is indeed something oboe-like in measures 21, 57, 63… and not in 14, 43, 67.… Then you decide to examine from the perspective of trumpets: the score tells you where there are trumpets, and your equipment shows trumpet wave forms at the specified measures. Eventually, if you do this from a large enough sample of instrumental perspectives and a large enough sample of measures, and make ever more close distinctions (as between oboe and English horn), and use ever more sophisticated equipment, because the original recording was made without *particular* regard for the particular perspectives that you have sampled, you will eventually conclude that the recording is of high fidelity *even though you yourself cannot have any direct access to the fidelity of the recording itself*.

By analogy, if apprehensions/descriptions of pristine experience are indeed of high fidelity. It should be possible to examine them from a variety of perspectives not specifically intended in the original data gathering. If those not-specifically-intended examinations show expected characteristics, then we should take that as evidence that the original apprehensions/descriptions were of high fidelity (In passing, we note that it is the radically non-subjective nature of pristine inner experience that makes this kind of multiple-perspective exploration possible). To explore the putative fidelity of DES apprehensions/descriptions, we proceeded in two basic steps.

First, time-locked to recordings of brain activation using fMRI, we used DES to apprehend and then describe ongoing experience in (putative) high fidelity—that is, we aspired to faithful apprehensions/descriptions of phenomena as they present themselves of themselves (Hurlburt, 2011), *not* skewed or distorted. (Toward this end, the DES procedure and its expositional interviews are **“open-beginninged”** Hurlburt, 2011; Hurlburt et al., 2015; that is, the procedure does not specify in advance the feature(s) of inner experience to be investigated. Open-beginninged-ness is a necessary feature of fidelity, by analogy to the audio recording—the recording does not try, a priori, to record the *oboes* in high fidelity, it tries to record the *audio scene as it naturally occurs* in high fidelity, which can later be listened to for any features of interest, including oboes, trumpets, etc.). The procedure is described in Hurlburt et al. (2015), Kühn et al. (2014), and Hurlburt et al. (2016), and sketched briefly here. We trained participants in 4 days of DES sampling in the participant's natural environment, each with its attendant 1-h expositional (“iterative”) interview, which involved multiple co-interviewers. Thereafter each participant underwent nine 25-min fMRI scanner sessions, receiving four quasi-random DES beeps. Brain activations time-locked to those beeps were recorded. In the usual DES procedure, the co-interviewers wrote and edited a “contemporaneous” description of each of the sampled experiences from that session. This resulted, for each participant, in 9 × 4 = 36 beeped attempts to apprehend in-scanner inner experience, 36 written descriptions thereof, and 36 time-locked fMRI brain activations. There were five such participants, resulting in a total of 5 × 36 = 180 experiences/activations.

KEY CONCEPT 7 **Open-beginninged**

Exploring whatever phenomena present themselves rather than starting with predefined interest; a fundamental part of the bracketing of presuppositions. The question “What, if anything, was in your experience at the moment of the beep?” is open-beginninged because it invites apprehension of any phenomena without favoring inner speech, imagery, sensation, or any other particular kind of experience.

The second part of our exploration of fidelity involves, by analogy from our deaf engineer, examining the apprehensions/descriptions (obtained in the first part) from a variety of perspectives not explicitly contemplated during data collection. For example, if the interviews happen to describe inner speaking as being ongoing at beep 7, 16, 29, 31, 84, 93, and 142 but not at the remaining beeps, and the brain activations modeled on those particular inner-speaking beeps differ from activations modeled on the remaining beeps in ways relevant to speech, we have one bit of evidence in favor of fidelity of apprehension/description. If the interviews happen to describe visual imagery as being ongoing at particular beeps but not at the remaining beeps, and the brain activations so modeled show characteristics relevant to vision, we have another bit of evidence for fidelity.

Kühn et al. (2014) explored one such perspective by noting that the expositional interviews of one of their participants, “Lara,” indicated that eight out of 36 samples included inner speaking. fMRI analysis on this individual showed that the eight inner-speaking samples were indeed accompanied by increased activity in left inferior frontal gyrus (IFG), a main element of the speech network established by other fMRI studies. That result can be interpreted as a bit of evidence in favor of the credible fidelity of the apprehension/description of Lara's pristine experience because, during the sampling procedure, we had not been especially interested in Lara's inner speaking. When the expositional interviews identified eight in-scanner moments that happened to involve inner speaking, we could make a risky prediction that brain activation relevant to speaking had been ongoing at those moments: a prediction that was subsequently confirmed. Such evidence is not conclusive: it is possible, for example, that Lara had been speaking aloud at those moments but denied it in the interviews. Replications are required to distinguish among such possibilities.

Fidelity in general involves the potential for refinement of detail, and this study allowed such refinement with respect to inner speaking. Lara's experience, as putatively revealed in the expositional interviews, included a continuum of the manner in which she apprehended inner words, ranging from innerly spoken to innerly heard. Lara's brain activity during those moments claimed to be of inner speaking, when contrasted to moments claimed to be innerly hearing her own voice, showed increased activity in left IFG. This is another bit of evidence in favor of the credibility of the fidelity of the DES apprehensions/descriptions.

Hurlburt et al. (2016) re-examined these data (including data of five participants) from a somewhat different perspective. On the first day of participation in the study (and prior to any DES involvement, participants had been placed in the scanner and asked to complete five typical in-scanner tasks such as: form a mental image of a pencil, imagine hearing a tinkling, feel anxiety, feel a shiver, and innerly say “elephant.” Then DES training and sampling in the scanner was performed as described above. Hurlburt et al. showed that of the 180 in-scanner samples across all participants, the expositional interviews identified 52 that involved spontaneous inner speaking (recall that this study was open-beginninged—we had not specifically targeted inner speaking). The brain activation that had been recorded during those 52 moments could be compared to the brain activation that had been recorded from the same participants during the task-elicited inner speech (e.g., say “elephant”). Whereas task-elicited inner speech was associated with decreased activation in Heschl's gyrus (and also left IFG increase), spontaneous inner speech was associated with *increased* Heschl's gyrus activation. That was surprising because Heschl's gyrus is a brain area usually understood to be involved in hearing. Because activations in a targeted brain region differentiated between task-elicited and spontaneous inner speaking, this result can be interpreted as another bit of evidence in favor of the fidelity of apprehension/description of pristine (spontaneous) experience.

Recalling that fidelity involves the potential for refinement of detail, the putative fidelity of the descriptive procedure allowed the investigators to notice that some of those 52 moments of inner speaking also (simultaneously) involved prominent inner characteristics *not* related to speaking (visual imagery, for example), and other samples were of moments where the participant and/or the interviewers were not confident that inner speaking had been ongoing. Those samples could be removed from the analysis, resulting in 20 samples where the investigators were confident that inner speaking was the most prominent aspect. The fMRI analysis was repeated for those 20 samples, with results similar to the 52-moment results. This is another bit of evidence in favor of the fidelity of apprehension/description of pristine experience.

In sum: if apprehensions/descriptions of inner experience are indeed of high fidelity, then it should be possible to “mine” those apprehensions/descriptions from a variety of perspectives not explicitly considered when the apprehensions/descriptions were created. For one example, Smallwood and colleagues (Smallwood, 2011; Smallwood et al., 2012) have proposed that the fluctuation between task-centered cognition and mind wandering involves switching between neural networks that process the externally imposed environmental task and different networks that process internally generated information. If the Hurlburt et al. (2016) apprehensions/descriptions are of high fidelity, it should be possible to re-examine their samples from an internal/external perspective and then determine whether the corresponding brain activations match Smallwood's theoretical predictions. A similar process could be undertaken for any theory that claims a link between experience and brain activity.

## Discussion

We have made the case that apprehending and describing inner experience in high fidelity is important to science, and therefore that it will be necessary for science to figure out how to evaluate the credibility of claims about fidelity. We have discussed one potential avenue for evaluating such claims—examining fMRI data from multiple experiential perspectives not originally contemplated when the data were collected. We intend this discussion to be a small step in an important direction, more about raising potentialities than of establishing results, but we suggest there are enough bits of evidence to suggest that the fidelity of apprehensions/descriptions can be productively explored by examining and re-examining pairings of moments of experience and brain activations in multiple ways from multiple perspectives.

The DES studies described here are expensive in terms of time, expertise, and equipment. It is reasonable to ask whether such studies are worth science's effort. It seems to us that fundamental principles are at stake. High fidelity apprehensions/descriptions of experience are necessary to examine claims that form the basis of consciousness science (such as that inner speech is ubiquitous), are important in advancing science's understanding of brain function (such as that inner speaking and inner hearing have different neural signatures), and may be useful in refining constructs that have been suggested by other programmes of research (such as Smallwood's internal/external theory).

Hurlburt et al. (2016) suggest another implication of high-fidelity data collection. The power of a statistical test is essentially the effect size times the sample size divided by the experimental error. Most fMRI studies attain adequate power by using a large sample size to increase the numerator. However, it may also be possible (as described above) to attain adequate power by selecting more experientially homogeneous samples to decrease the denominator experimental error. It was the high fidelity data collection that made it possible to notice that of the 52 samples that included inner speaking, only 20 involved inner speaking as the most salient characteristic. It was then possible to use only those 20 samples, thus making the experiences more homogeneous and thereby reducing the experimental error. Such refinement would most likely not be possible without high fidelity apprehensions/descriptions.

Many observers have suggested the desirability of versions of DES that involve less time and less expertise (e.g., Froese et al., 2011; Alderson-Day and Fernyhough, 2015; McAuliffe and McGann, 2016). Alternatively, it might be observed that fidelity considerations suggest the desirability that science spend more of its resources in cultivating methods that seek to provide high fidelity observations. A mature science of experience would work through the situations in which each would be desirable.

It is not our intention to contend that DES is the epistemic tribunal against which all methods of introspection should be judged (Hurlburt and Schwitzgebel, 2011). Other traditions have also shown the usefulness of combining disciplined first-person approaches with neuroscience, for example the neurophenomenology of Varela (1996) and colleagues, the visual perception studies of Lutz et al. (2002), the seizure anticipation experience of Petitmengin et al. (2006), and the expectation explorations of Price and Barrell (2012). Our aim is to encourage discussion of first-person fidelity and the criteria for establishing it.

## Author contributions

All authors participated in the original article data collection and writing. All authors participated in the writing of this manuscript.

### Conflict of interest statement

The authors declare that the research was conducted in the absence of any commercial or financial relationships that could be construed as a potential conflict of interest.
